# Association Between Diet Quality and 20-Year Heart Failure Incidence: Findings from the ATTICA Cohort Study (2002–2022)

**DOI:** 10.3390/nu18193278

**Published:** 2026-10-06

**Authors:** Christina Chrysohoou, Panagiotis Iliakis, Ioana Christina Daha, Ioannis Leontsinis, Kyriaki Mavromoustakou, Evangelia Damigou, Konstantinos Konstantinou, Christos Pitsavos, Konstantinos Tsioufis, Demosthenes B. Panagiotakos

**Affiliations:** 1 First Cardiology Clinic, Hippokration Hospital, Medical School, National and Kapodistrian University of Athens, 15772 Athens, Greece; panayiotisiliakis@gmail.com (P.I.); giannisleontsinis@gmail.com (I.L.); mavromoustakoukiriaki@yahoo.gr (K.M.); cpitsavo@med.uoa.gr (C.P.); ktsioufis@gmail.com (K.T.); 2 Cardiology Department, Carol Davila University of Medicine and Pharmacy, 050474 Bucharest, Romania; dahaioana@gmail.com; 3 Department of Nutrition and Dietetics, School of Health Sciences and Education, Harokopio University, 17676 Athens, Greece; edamigou@hua.gr (E.D.); dbpanag@hua.gr (D.B.P.)

**Keywords:** Global Diet Quality Score, diet quality, heart failure, dietary patterns, prospective cohort, Mediterranean population

## Abstract

**Background/Objectives:** Diet quality is a potentially modifiable determinant of heart failure (HF), yet evidence regarding its long-term association with HF incidence remains limited. This study examined the association between baseline diet quality and 20-year incident HF in the population-based ATTICA cohort. **Methods:** Among 3042 adults free of cardiovascular disease at baseline (2001–2002), HF status was available for 2159 participants in 2022, of whom 241 (11.2%) developed HF. Diet quality, including the relative contributions of healthy and unhealthy food groups, was assessed using the Global Diet Quality Score (GDQS), derived from a validated food-frequency questionnaire. Logistic regression models were sequentially adjusted for age, sex, body mass index, education, physical activity, smoking burden, hypertension, diabetes, and hypercholesterolemia. **Results:** HF incidence was 20.7%, 5.1%, and 6.6% among participants with low, intermediate, and high diet quality, respectively (*p* < 0.001). In the fully adjusted analysis (n = 1973), better overall diet quality was associated with lower odds of HF (odds ratio [OR] per 1-point increment, 0.97; 95% confidence interval [CI], 0.94–0.99). A greater contribution of healthy foods was also inversely associated with HF (OR, 0.96; 95% CI, 0.93–0.98), whereas the unhealthy-food component was not independently associated with the outcome (OR, 1.04; 95% CI, 0.96–1.13). **Conclusions:** Better diet quality, particularly greater consumption of health-promoting foods, was independently associated with lower 20-year HF incidence in a Mediterranean population.

## 1. Introduction

Heart failure (HF) is a major and increasing public health burden, driven by population ageing, improved survival after cardiovascular disease (CVD), and the accumulation of cardiometabolic risk factors [1,2]. Because HF is commonly preceded by hypertension, obesity, diabetes, dyslipidemia, and coronary disease, prevention requires attention to modifiable exposures long before clinical symptoms develop. Diet may play a particularly important role in HF prevention because it can simultaneously influence several interrelated pathways involved in its development. A high-quality dietary pattern may support blood-pressure control, healthy body weight, glucose regulation, and a favorable lipid profile while attenuating systemic inflammation, oxidative stress, and endothelial dysfunction. These effects may reduce the cumulative burden of hypertension, obesity, diabetes, coronary heart disease, and other major antecedents of HF. Consequently, improving overall diet quality represents a comprehensive preventive strategy rather than an intervention targeting a single cardiovascular risk factor [3].

Robust prospective evidence examining the association between overall dietary patterns and long-term incident HF remains comparatively limited and less extensive than that available for broader CVD outcomes or type 2 diabetes. Greater adherence to the Dietary Approaches to Stop Hypertension (DASH) pattern has been associated with lower HF incidence in large cohorts [4,5], although findings have not been entirely consistent across populations [6]. These differences may reflect variation in dietary assessment, background diet, population risk, follow-up duration, and the degree to which a score captures both protective and adverse foods.

Over the past decades, nutritional epidemiology has increasingly focused on the study of dietary patterns, with the Mediterranean diet being among the most extensively investigated. Although such patterns may capture important dimensions of diet quality, they primarily assess adherence to a predefined combination of foods and may not fully reflect the overall nutritional adequacy and healthfulness of an individual’s diet. Their applicability may also depend on cultural context, while the use of population-specific scoring thresholds can limit comparisons across studies [7]. Diet quality therefore represents a broader construct that considers the balance, diversity, and adequacy of both health-promoting and less favorable foods, without requiring adherence to a particular regional dietary pattern. Unlike pattern-specific indices, diet-quality measures do not require an individual’s food intake to conform to a predefined or culturally specific eating model. The Global Diet Quality Score (GDQS) is a food-based metric developed to assess these dimensions across diverse populations and geographical settings. Its 25 food groups jointly capture nutrient adequacy and diet-related noncommunicable disease risk. The total score ranges from 0 to 49 and comprises a healthy-food component (GDQS+; range 0–32) and an unhealthy-food component (GDQS−; range 0–17), with higher values indicating a more favorable dietary profile [7]. Although Mediterranean diet scores are well validated and particularly relevant to Mediterranean populations, they primarily quantify adherence to a predefined regional pattern rather than the broader healthfulness of the diet. Moreover, several Mediterranean diet scores use population-specific cut-off points, which may limit cross-study comparability and reduce discrimination within populations where Mediterranean-type dietary habits are relatively common. The GDQS therefore provides a standardized assessment of both health-promoting and less favorable food consumption and facilitates the international comparison of findings. Previous studies have associated the GDQS with nutrient adequacy, metabolic syndrome, type 2 diabetes, and cardiovascular outcomes [8,9,10]; however, evidence regarding its association with long-term HF incidence remains limited.

The present study therefore evaluated baseline GDQS, GDQS+, and GDQS- in relation to 20-year incident HF among adults initially free of CVD in the ATTICA population-based cohort. It was hypothesized that higher overall GDQS and higher GDQS+ would be associated with lower HF odds after accounting for demographic, lifestyle, and clinical risk factors.

## 2. Materials and Methods

### 2.1. Study Design

The ATTICA study is a prospective, population-based cohort of Greek adults with baseline examination in 2001–2002 and follow-up assessments at 5, 10, and 20 years. The study design and baseline procedures have been described previously [11,12]. The protocol conformed to the Declaration of Helsinki and was approved by the institutional Ethics Committees of the University of Athens Medical School (#017/1.5.2001) and Harokopio University (#38/29.03.2022). All participants provided written informed consent.

### 2.2. Setting and Participants

**Figure 1:**
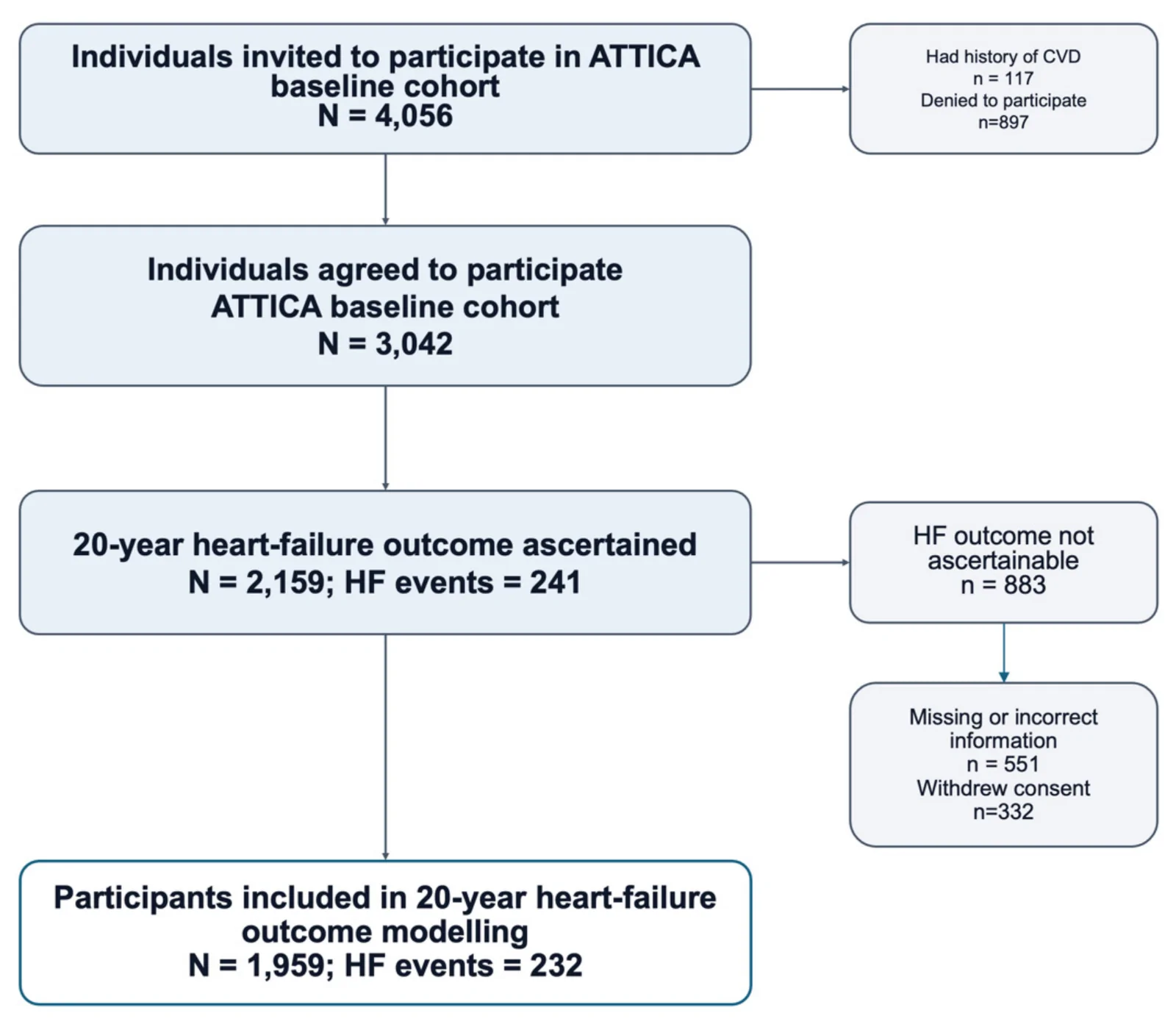
Participant flow and analytic samples.

A total of 4056 adults living in the Attica region of Greece were randomly invited between May 2001 and August 2002, using sampling stratified by sex, age group, and area of residence. Of these, 3042 adults fulfilled the inclusion criteria, agreed to participate and were enrolled. Individuals aged below 18 years, or with established CVD, cancer or any inflammatory disease at baseline were excluded according to the study protocol. For the present analysis, 2159 participants had an ascertainable 20-year HF status and constituted the analytic cohort; 883 participants without recorded 20-year HF status were not included in the outcome analyses (Figure 1). No significant differences were observed between participants retained at follow-up and those lost with respect to age, sex, smoking habits, or the distribution of major cardiovascular risk factors studied here (all *p*-values > 0.20). However, participants lost to follow-up had a higher educational level than those who remained in the study (*p* < 0.03).

### 2.3. Baseline Measurements

#### 2.3.1. Sociodemographic and Clinical Characteristics

Among several socio-demographic factors recorded at baseline examination, age (in years), sex, and years of education were used in the present analysis. From the anthropometric characteristics, body weight and height were measured; body mass index (BMI) was calculated as body weight in kilograms divided by height in meters squared. Clinical status assessment included history of hypertension defined as systolic blood pressure ≥140 mmHg, diastolic blood pressure ≥90 mmHg, or use of antihypertensive medication; type 2 diabetes defined as fasting plasma glucose ≥126 mg/dL or use of glucose-lowering medication; and hypercholesterolemia, defined as total cholesterol >200 mg/dL or lipid-lowering treatment. Family history of CVD was also recorded. Details about the procedures followed may be found in previous publications [11].

#### 2.3.2. Lifestyle Characteristics

Lifestyle characteristics were also recorded [12]. Physical activity was assessed with the validated short-form International Physical Activity Questionnaire [13]. Participants classified above the minimally active category were considered physically active for the present analyses. Smoking burden was expressed as pack-years, calculated from smoking duration and amount. Pack-years were analyzed only when recorded in the dataset; no unverified assumptions were made for missing values.

#### 2.3.3. Diet Quality Assessment

Habitual dietary intake was assessed at baseline with the validated 156-item semi-quantitative EPIC-Greek food-frequency questionnaire, administered with assistance from trained dietitians [14]. Food intake was mapped to the 25 GDQS food groups and scored according to published methods [7,8]. Higher GDQS values represent better overall diet quality. GDQS+ assigns higher values for greater intake of health-promoting food groups, whereas higher GDQS- values represent lower or more favorable intake of food groups considered unhealthy or unhealthy when consumed in excessive amounts [7]. GDQS tertiles were also calculated (tertile 1, ≤35; tertile 2, >35 to ≤39; tertile 3, >39); unequal group sizes resulted from tied score values.

### 2.4. Twenty-Year Heart Failure Outcome

At the 20-year follow-up in 2022, study physicians ascertained, among other clinical outcomes, incident HF through participant evaluation and review of available medical records. HF diagnoses were classified according to the International Classification of Diseases, 10th Revision (I50.x). Information on exact dates of the establishment of HF phenotype was not available, as the follow-up protocol documented HF occurrence without systematically recording the timing of diagnosis or classifying cases according to left ventricular ejection fraction. Consequently, time-to-event analyses and separate evaluation of HF with preserved, mildly reduced, or reduced ejection fraction could not be performed. For participants who died before the 20-year examination, HF status was considered ascertainable only when an HF diagnosis before death was documented through the available medical, hospital, or mortality records. Deaths without documented evidence of HF were treated as having an unavailable HF outcome and were excluded from the outcome-ascertained sample.

### 2.5. Statistical Analysis

Continuous variables are presented as mean (standard deviation) when approximately normally distributed and as median (interquartile range) otherwise; categorical variables are presented as frequencies and percentages. Baseline differences across GDQS tertiles were evaluated using one-way analysis of variance, the Kruskal–Wallis test, or the chi-square test, as appropriate. HF cumulative incidence was calculated as the number of ascertained events divided by the number of participants in the cohort; proportions across tertiles were compared with the chi-square test, followed by Bonferroni-corrected pairwise comparisons. Because exact HF event times were unavailable, binary logistic regression was used to estimate odds ratios (ORs) and their corresponding 95% confidence intervals (CIs) for 20-year HF. GDQS, GDQS+, and GDQS- were modeled separately per 1-point increase; total GDQS was also modeled categorically, with tertile 1 as the reference. Model 1 adjusted for age (per 10 years) and sex. Model 2 additionally adjusted for BMI, education, physical activity, and pack-years. Model 3 additionally adjusted for baseline hypertension, diabetes, and hypercholesterolemia. Potential non-linearity was assessed using restricted cubic spline logistic regression with knots at the 10th, 50th, and 90th GDQS percentiles. The model included the fully adjusted covariates, used the median GDQS as the reference, and assessed non-linearity by likelihood-ratio testing. A sensitivity model further adjusted for family history of cardiovascular disease. Complete-case analysis was used within each model, and model-specific sample sizes are reported. All tests were two-sided, with *p* < 0.05 considered statistically significant. All statistical analyses were performed using STATA statistical software (version 17; College Station, TX, USA).

## 3. Results

### 3.1. Participant Flow and Baseline Characteristics

During the 20-year follow-up, 241 of the 2159 participants developed HF, corresponding to a cumulative incidence of 11.2%; of these cases, 108 (10.1%) occurred among men (*p* for sex differences = 0.143).

The median GDQS was 37.0 (interquartile range 23.9–37.0); the corresponding medians were 31.0 (14.0–31.0) for GDQS+ and 6.0 (6.0–6.0) for GDQS-. Participants in the lowest GDQS tertile were older, more often men, and had higher BMI and a greater prevalence of hypertension, diabetes, and hypercholesterolemia than participants in the higher tertiles. Physical activity was most frequent in the highest tertile of diet quality, whereas family history of CVD did not differ materially across tertiles (Table 1).

### 3.2. Heart Failure Incidence and Diet Quality

**Figure 2:**
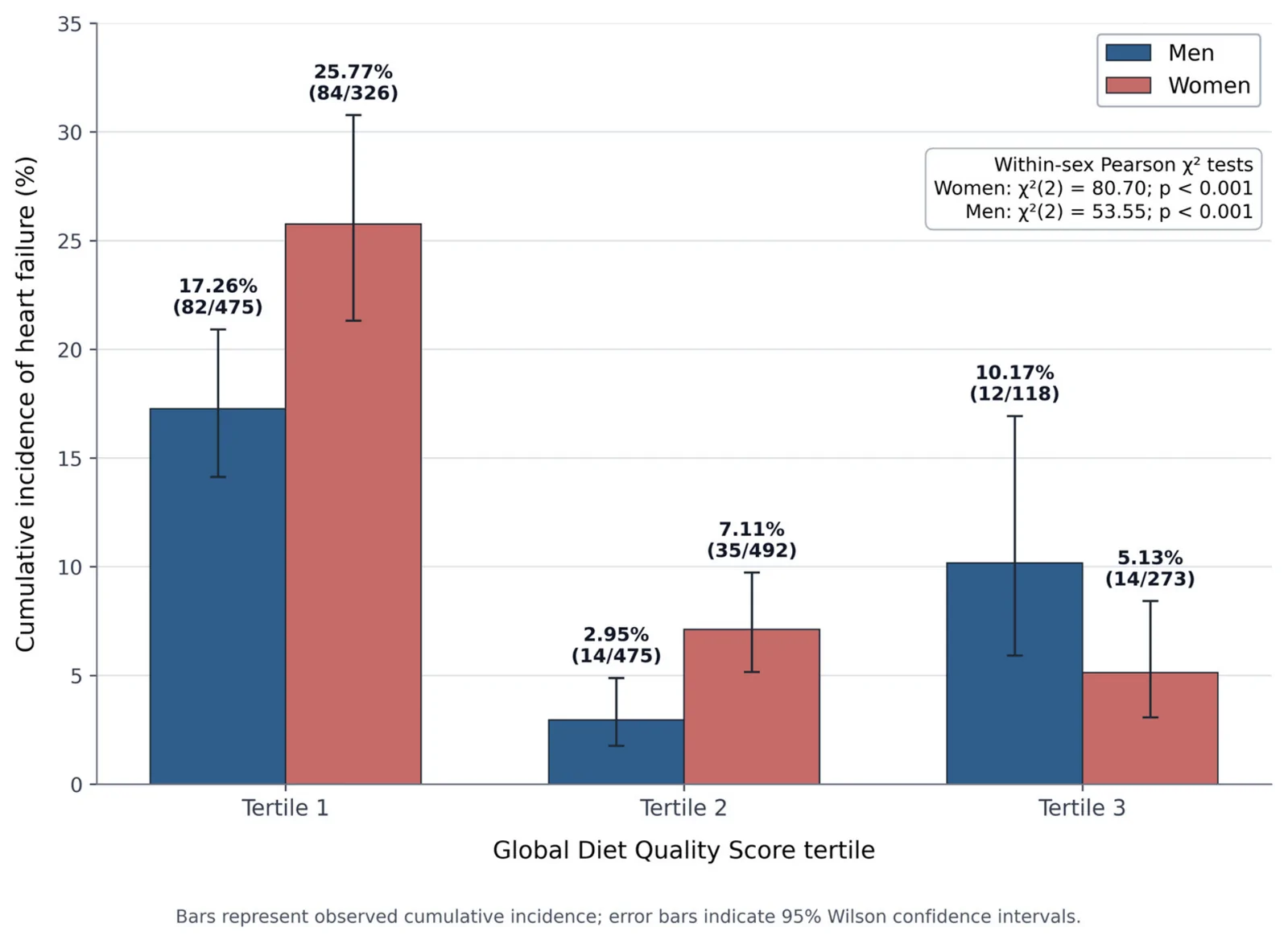
Twenty-year heart failure incidence according to Global Diet Quality Score tertile. Values are events/N (%). The global *p*-value was obtained using the chi-square test.

A strong association was observed between HF incidence and GDQS tertile (*p* < 0.001) (Figure 2). The cumulative incidence was 20.7% in tertile 1, 5.1% in tertile 2, and 6.6% in tertile 3 (Table 2). Bonferroni-corrected pairwise comparisons were significant for tertile 1 versus tertile 2 and tertile 1 versus tertile 3 (both *p* < 0.001), but not for tertile 2 versus tertile 3 (*p* = 0.916). A non-monotonic pattern in unadjusted HF proportions across GDQS tertiles was observed in men (*p* for non-linearity < 0.001), but not in women (*p* = 0.837).

### 3.3. Multivariable Associations

Despite the strong associations observed in the unadjusted analyses, potential confounding could not be excluded; therefore, multivariable analyses were subsequently performed to account for relevant covariates. After adjustment for age and sex, higher GDQS and GDQS+ were associated with lower HF odds, whereas GDQS- was not (Table 2). Associations for GDQS and GDQS+ remained significant after additional adjustment for BMI, education, physical activity, pack-years, hypertension, diabetes, and hypercholesterolemia. In Model 3, each 1-point higher GDQS was associated with 3% lower HF odds (*p* = 0.015), and each 1-point higher GDQS+ was associated with 4% lower odds (*p* = 0.003). GDQS- remained unassociated with HF (*p* = 0.354).

Relative to tertile 1, adjusted HF odds were lower in tertile 2 (*p* = 0.045) and tertile 3 (*p* = 0.002) in Model 3. After further adjustment for family history of CVD, the association for tertile 3 persisted (*p* = 0.016). The continuous GDQS association in this sensitivity Model 4 was borderline retained (*p* = 0.067); GDQS+ remained significant (*p* = 0.042). However, the models including BMI, hypertension, diabetes, and hypercholesterolemia should be considered as a secondary analysis of whether the association persisted beyond established risk factors, rather than as an estimate of the total causal association.

### 3.4. Diet Quality and 20-Year Heart Failure Risk by Cardiovascular Risk-Factor Burden

HF incidence increased progressively with cardiovascular risk-factor burden, in an exploratory analysis performed, from 3.3% among participants without any of the four risk factors to 8.8%, 15.6%, and 24.3% among those with one, two, and at least three risk factors, respectively (*p* < 0.001).

**Figure 3:**
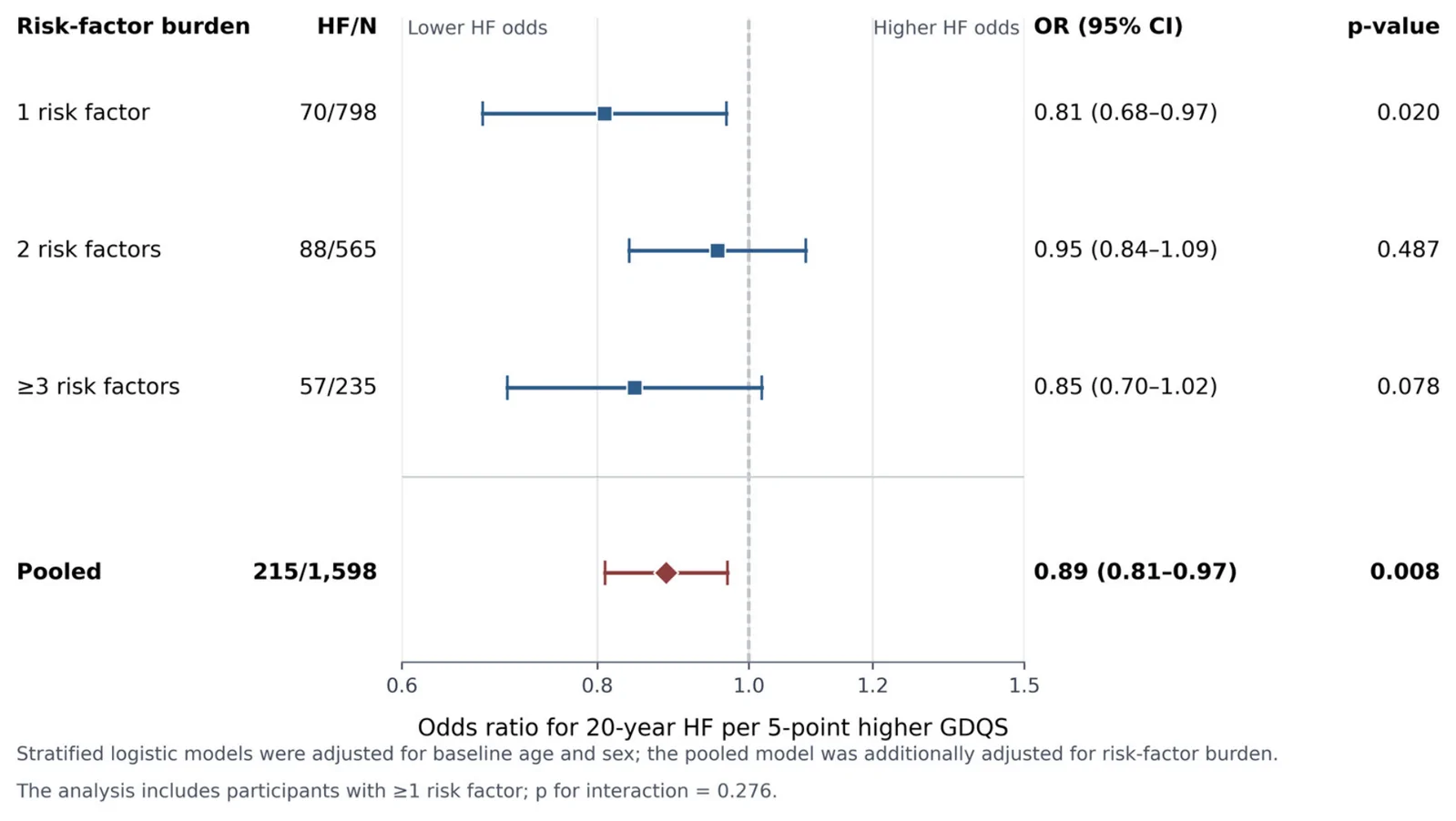
Twenty-year heart failure incidence according to Diet Quality, by Cardiovascular Risk Factor Burden.

In age- and sex-adjusted analyses, higher GDQS was significantly associated with lower HF odds among participants with exactly one cardiovascular risk factor. Each 1-point higher GDQS was associated with a 4% reduction in HF odds (OR 0.96, 95% CI 0.92–0.99; *p* = 0.020), corresponding to an OR of 0.81 per 5-point increment (Figure 3). However, among participants with at least two or three cardiovascular risk factors, the association was inverse but did not reach statistical significance. In the pooled analysis additionally accounting for risk-factor burden, each 1-point higher GDQS was associated with lower HF odds (OR 0.98, 95% CI 0.96–0.99; *p* = 0.007). A sensitivity model incorporating a quadratic age term and a bias-reduced logistic analysis yielded similar results.

## 4. Discussion

In this population-based Mediterranean cohort, approximately one in nine participants developed HF during the 20-year follow-up, without any sex differences, underscoring the substantial burden of HF arising within the community among individuals initially free of CVD. This finding is particularly relevant given the growing contemporary burden of HF associated with population ageing, prolonged exposure to cardiometabolic risk factors, and improved survival from other cardiovascular conditions. The importance of long-term cardiometabolic risk accumulation is further supported by recent 20-year findings from the ATTICA cohort in postmenopausal women, in whom diabetes and hypercholesterolemia emerged as significant predictors of cardiovascular events [15]. The study adds novel long-term evidence to an area in which research has focused considerably more on coronary disease, stroke, and diabetes than on incident HF. Higher baseline diet quality was independently associated with lower HF incidence after accounting for demographic, lifestyle, and clinical risk factors, suggesting that dietary healthfulness may contribute information beyond established HF determinants. When the two GDQS submetrics were examined separately, the inverse association was primarily reflected by GDQS+, the healthy-food component representing greater consumption of health-promoting food groups, whereas GDQS−, the component reflecting consumption of less favorable food groups, was not independently associated with HF. Collectively, these findings extend the relevance of diet quality to long-term HF prevention and highlight the potential importance of promoting nutrient-rich, health-supporting foods as part of community-based strategies to reduce the growing HF burden.

The association between diet and HF incidence has been studied before in some population-based cohort studies from the US, Europe, China, and some other parts of the world [2,3,4]. The presented findings extend existing evidence linking healthful dietary patterns with HF prevention. In Swedish cohorts, long-term adherence to the DASH diet was associated with lower HF risk, and replacing red or processed meat with foods emphasized by DASH was associated with additional benefit [4]. A US cohort similarly reported an inverse association between DASH adherence and incident HF [5], whereas other studies have produced null or opposing estimates [6]. More recently, a UK Biobank analysis found lower HF incidence or HF-related death among participants with greater DASH adherence [16]. However, the use of *a priori* (predefined) dietary patterns involves several methodological constraints. The meaning and performance of these patterns depend heavily on decisions regarding component selection, intake thresholds, scoring direction, weighting, energy adjustment, and aggregation. Most indices also assume that dietary components exert independent, additive, and often equally weighted effects, although this may not reflect their biological relationships. Furthermore, the same total score can arise from substantially different combinations of foods because favorable intake in one component may compensate for unfavorable intake in another. Predefined scores are also constrained by the nutritional evidence and dietary recommendations available when they are developed and may therefore omit relevant dimensions of diet quality. Consequently, observed associations may partly reflect the architecture and discriminatory capacity of the selected index, rather than solely the underlying relationship between dietary intake and disease [17,18,19,20,21].

The component-specific findings provide further insight into which dimension of diet quality may be most relevant to long-term HF development. Although the overall GDQS integrates both health-promoting and less favorable food groups, the observed association appeared to arise predominantly from greater consumption of healthy foods. In contrast, the unhealthy-food component was not independently associated with HF, indicating that increasing health-promoting foods and limiting unfavorable foods may not represent biologically equivalent dimensions of dietary improvement [22]. This distinction may also reflect differences in the range, frequency, or measurement accuracy of the foods included in each component. The stronger association for GDQS+ is biologically plausible. Diets rich in fruits, vegetables, whole grains, legumes, nuts, and fish can improve blood pressure, glycemic regulation, body weight, lipid profiles, endothelial function, and systemic inflammation, thereby acting on multiple upstream HF pathways [3,23,24]. These mechanisms may be particularly relevant in the setting of obesity, in which metabolic dysregulation, systemic inflammation, and altered volume homeostasis interact to promote the development and progression of HF [25]. The persistence of the association after adjustment for hypertension, diabetes, hypercholesterolemia, BMI, and smoking burden suggests that these measured intermediates did not fully account for the observed relationship. Nevertheless, mediation cannot be established from the available data, and residual confounding by other socioeconomic, behavioral, or clinical characteristics remains possible. The absence of an association for GDQS- should not be interpreted as evidence that foods classified as unhealthy are harmless. GDQS- had limited variability in this cohort. Moreover, a score emphasizing the addition of beneficial foods may perform differently in a Mediterranean setting, where background dietary habits and food combinations differ from those in populations used for initial validation. Other GDQS studies have also suggested that the relative contributions of healthy and unhealthy submetrics vary by outcome and population [8,9,10]. In 88,520 Nurses’ Health Study II participants followed from 1991 to 2017, 6305 developed type 2 diabetes. Women in the highest GDQS quintile had a 17% lower risk than those in the lowest. The association was consistent across age groups but driven mainly by lower unhealthy-food intake, and performed similarly to AHEI-2010 [9].

Despite effective pharmacotherapy, diet quality should remain central to HF prevention because it acts before structural cardiac damage becomes clinically evident. A diet rich in vegetables, fruits, whole grains, legumes, nuts, fish, and unsaturated oils, while limiting sodium, processed meat, refined carbohydrates, and added sugars, can simultaneously improve blood pressure, adiposity, insulin sensitivity, lipid metabolism, and renal sodium handling [26]. These changes may reduce left-ventricular hypertrophy, myocardial fibrosis, endothelial dysfunction, oxidative stress, systemic inflammation, and coronary atherosclerosis, interconnected processes contributing to both HF with preserved and reduced ejection fraction. High-quality dietary patterns may also lower the probability that hypertension, diabetes, obesity, and coronary disease accumulate over time, thereby reducing the substrate from which HF develops. Prospective evidence linking plant-based and DASH-style patterns with lower incident HF supports this preventive potential [27]. Accordingly, dietary improvement should complement risk-factor treatment throughout adulthood, emphasizing sustained overall eating patterns rather than isolated nutrients or short-term restriction [27].

### Strengths and Limitations

Strengths include the prospective, population-based design; inclusion of men and women initially free of CVD; a 20-year follow-up; dietary assessment by a validated FFQ administered with trained support; and physician-led outcome ascertainment supplemented by medical record review. The availability of GDQS submetrics also allowed the overall association to be separated into healthy- and unhealthy-food components.

Several limitations warrant consideration. First, GDQS was measured only at baseline and may not represent dietary changes over two decades. Second, FFQ-based assessment is subject to recall error and misclassification. Third, exact HF event dates and ejection-fraction phenotypes were unavailable, precluding time-to-event and subtype-specific analyses. Fourth, total energy intake, kidney function, atrial fibrillation, and other potential confounders were not available in the analytic dataset, raising the possibility of residual confounding. Fifth, some missing cases in variables included in models may have introduced selection bias. Moreover, the wide confidence intervals and attenuation of the continuous GDQS association in Model 4 indicate considerable uncertainty regarding the magnitude and independence of the observed relationship. These findings should therefore be interpreted cautiously and not solely according to conventional thresholds of statistical significance. Finally, the observational design precludes causal inference, and findings from adults living in Attica may not generalize to other populations.

## 5. Conclusions

In the population-based ATTICA cohort, higher baseline diet quality, particularly greater consumption of health-promoting foods, was associated with lower odds of incident HF during 20 years of follow-up. These findings support further investigation of the potential role of diet quality in HF development, including the biological pathways that may link dietary patterns with cardiac function. Given the substantial and increasing population burden of HF, clarifying the contribution of diet quality may help inform scalable public health strategies targeting modifiable dietary behaviors.

## Figures and Tables

**Table 1:** Baseline characteristics of participants according to Global Diet Quality Score tertile (n = 2159).

Characteristic	GDQS Tertile 1 (n = 801)	GDQS Tertile 2 (n = 967)	GDQS Tertile 3 (n = 391)	*p*-Value
Age, years	49.8 (13.9)	42.1 (10.6)	36.6 (15.3)	<0.001
Men, %	59.3	49.1	30.2	<0.001
Education, years	12.0 (9.0–16.0)	12.0 (12.0–16.0)	12.0 (12.0–16.0)	<0.001
Body mass index, kg/m^2^	27.4 (4.6)	25.7 (3.2)	24.6 (6.2)	<0.001
Hypercholesterolemia, %	45.9	43.0	24.4	<0.001
Hypertension, %	40.8	24.7	19.8	<0.001
Diabetes mellitus, %	12.0	3.4	3.8	<0.001
Family history of CVD, %	38.6	35.0	37.1	0.407
Physically active, %	35.6	35.0	43.2	0.012
Pack-years	24.0 (10.0–40.0)	16.0 (7.2–30.0)	10.2 (4.0–25.0)	<0.001

Values are mean (standard deviation), median (interquartile range), or percentage. *p*-values were obtained using one-way analysis of variance, the Kruskal–Wallis test, or the chi-square test, as appropriate. Pack-years are summarized among participants with recorded values. BMI, body mass index; CVD, cardiovascular disease. Missingness among participants with known HF status was 133 participants for hypertension (6.2%), 38 participants for education (1.8%), 31 participants for BMI (1.4%), and 1 participant for hypercholesterolemia (<0.1%); the remaining variables were complete.

**Table 2:** Associations of the Global Diet Quality Score and its submetrics with 20-year incident heart failure.

Exposure	Model 1	Model 2	Model 3	Model 4
N/events	2159/241	2096/241	1973/237	1959/232
GDQS, per 1-point increase	0.98 (0.96–0.99) *p* = 0.004	0.97 (0.94–1.00) *p* = 0.022	0.97 (0.94–0.99) *p* = 0.015	0.97 (0.94–1.001) *p* = 0.067
GDQS+, per 1-point increase	0.97 (0.95–0.99) *p* < 0.001	0.96 (0.93–0.99) *p* = 0.004	0.96 (0.93–0.98) *p* = 0.003	0.96 (0.93–0.99) *p* = 0.042
GDQS-, per 1-point increase	1.03 (0.98–1.08) *p* = 0.248	1.05 (0.97–1.13) *p* = 0.271	1.04 (0.96–1.13) *p* = 0.354	1.01 (0.90–1.12) *p* = 0.911
GDQS tertile 1	Reference	Reference	Reference	Reference
GDQS tertile 2 vs. tertile 1	0.67 (0.43–1.06) *p* = 0.090	0.45 (0.21–0.97) *p* = 0.040	0.46 (0.21–0.98) *p* = 0.045	0.56 (0.23–1.41) *p* = 0.220
GDQS tertile 3 vs. tertile 1	0.41 (0.19–0.86) *p* = 0.018	0.08 (0.02–0.38) *p* = 0.002	0.08 (0.02–0.37) *p* = 0.002	0.10 (0.01–0.64) *p* = 0.016

Values are odds ratios (95% confidence intervals) with two-sided p-values. Each exposure was entered in a separate model. Model 1: age (per 10 years) and sex. Model 2: Model 1 plus body mass index, education, physical activity, and pack-years. Model 3: Model 2 plus hypertension, diabetes mellitus, and hypercholesterolemia. Sensitivity Model 4: Model 3 plus family history of cardiovascular disease. Complete-case analysis was used within each model. GDQS, Global Diet Quality Score.

## Data Availability

The data are available from the corresponding author upon reasonable request. The data are not publicly available because they contain participant-level health information subject to privacy and ethical restrictions.
